# A Case Report and Global Perspective on Fusarium Superficial Keratitis in the Pediatric Population

**DOI:** 10.7759/cureus.92937

**Published:** 2025-09-22

**Authors:** S Avinash, Rajesh Kanna Kannabiran, Rahul Garg, Pravalika Rebbala, Sree Ranjani S, Siddharam S Janti, Shalam Nikhat Sheerin, Rahul Narang

**Affiliations:** 1 Department of Microbiology, All India Institute of Medical Sciences, Bibinagar, IND; 2 Department of Ophthalmology, All India Institute of Medical Sciences, Bibinagar, IND

**Keywords:** antifungal therapy, corneal ulcer, fungal keratitis, fusarium, pediatric infections

## Abstract

Fungal keratitis is a major cause of corneal blindness in tropical regions, with Fusarium species predominating. Pediatric cases, though less common, carry a greater risk of amblyopia and long-term disability. We report a nine-year-old boy who developed Fusarium keratitis after minor ocular trauma and unsupervised corticosteroid use. Corneal scrapings showed septate hyphae, and identification confirmed *Fusarium solani* by matrix-assisted laser desorption/ionization time-of-flight mass spectrometry. Treatment with topical natamycin and voriconazole led to complete healing without surgery. This case highlights the need for early recognition, rapid microbiological confirmation, and appropriate antifungal therapy in pediatric fungal keratitis, while underscoring the dangers of unsupervised corticosteroid use in endemic regions.

## Introduction

Fungal keratitis is a severe, vision-threatening corneal infection predominantly found in tropical and subtropical regions, where it accounts for up to 50% of corneal ulcers, particularly in South Asia and sub-Saharan Africa [1]. Among the causative pathogens, Fusarium spp. is recognized as one of the most virulent filamentous fungi associated with rapid progression and poor prognosis if not treated early [2]. While the clinical and microbiological characteristics of fungal keratitis in adults are well documented, pediatric presentations remain underreported and often underrecognized [3].

Children are susceptible to corneal infections due to their frequent exposure to environmental agents and a delay in reporting symptoms. Diagnosis is further complicated by nonspecific early signs, potential difficulty in patient cooperation during examination, and the tendency for delayed referral [4]. Moreover, the unsupervised use of over-the-counter corticosteroid eye drops can suppress immune responses and worsen fungal infections, making timely identification and appropriate therapy crucial [5].

Here, we report a rare pediatric case of Fusarium superficial keratitis with clinical features and rapid microbiological confirmation. The case was successfully treated with dual topical antifungal therapy. This report aims to contribute to the limited pediatric literature and reinforce the importance of early empirical management in suspected fungal keratitis.

## Case presentation

A nine-year-old boy presented to the ophthalmology outpatient services in an tertiary care hospital with a two-day history of redness, ocular pain, photophobia, and blurring of vision in the left eye following minor trauma with a foreign body sustained during play. Before presentation, he had self-administered over-the-counter corticosteroid eye drops. There was no history of systemic or ocular comorbidities, and baseline viral markers were negative.

**Figure 1:**
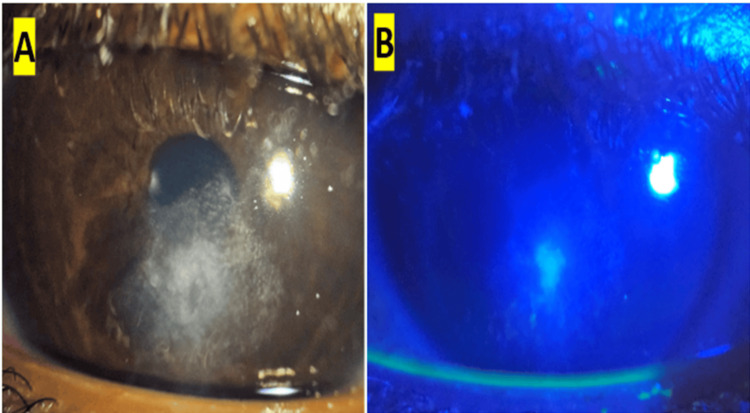
Slit‑lamp examination of the left eye (A) Slit‑lamp image showing a 1‑mm central epithelial defect with surrounding stromal edema and peripheral scarring. (B) Cobalt blue illumination with fluorescein staining highlighting the epithelial defect

On examination, he was alert, afebrile, and systemically stable. Uncorrected visual acuity was 6/6 in the right eye and 6/12 in the left eye, improving with pinhole. Slit-lamp evaluation of the left eye revealed a central, dry corneal ulcer showing a 1-mm epithelial defect with surrounding stromal edema and peripheral scarring (Figure 1A). Fluorescein staining demonstrated a 1-mm epithelial defect (Figure 1B). Intraocular pressure and lens status were within normal limits. The posterior segment could not be visualized due to corneal opacity.

**Figure 2:**
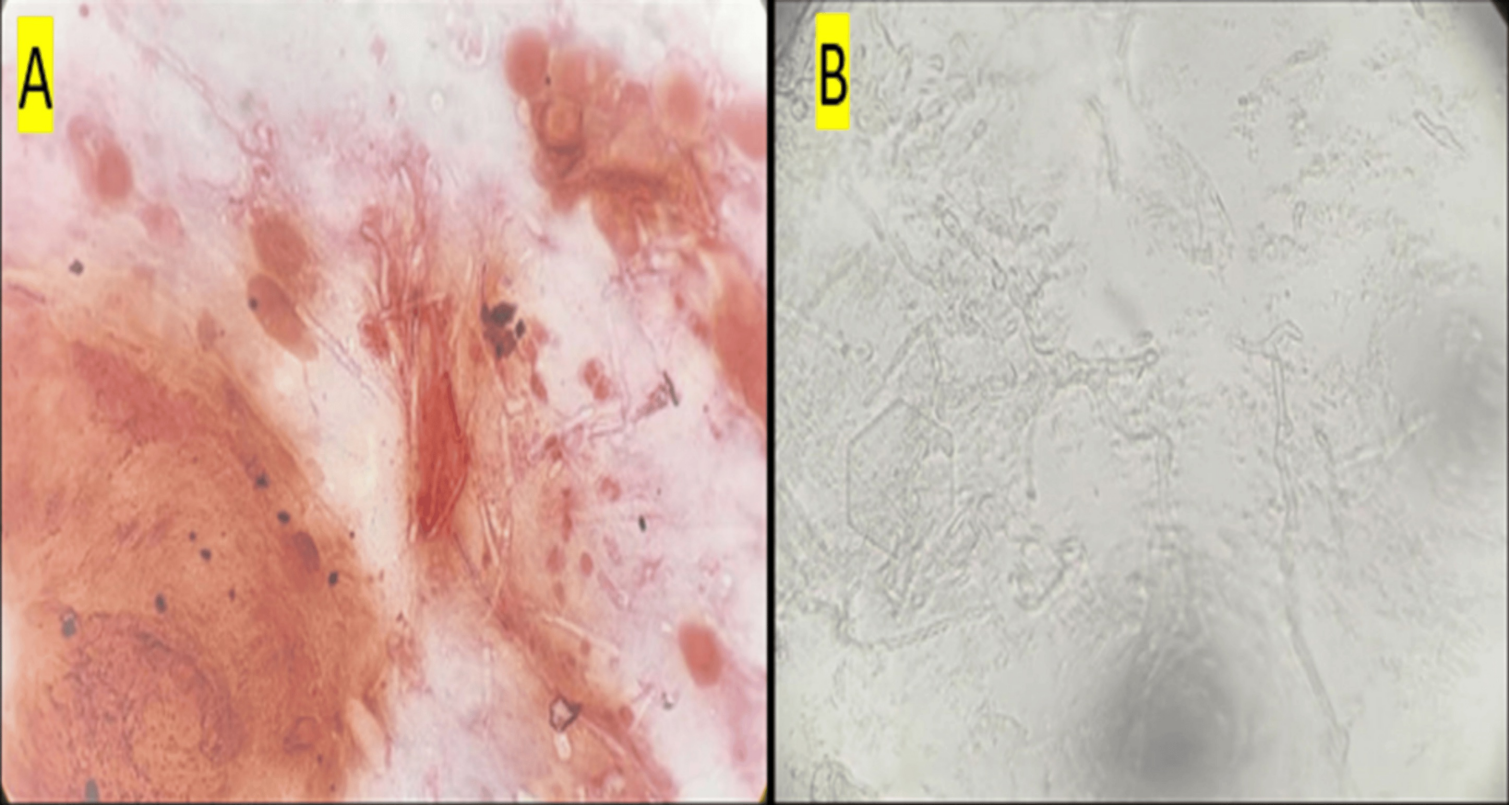
Direct microscopy of corneal scrapings (A) Gram stain showing faintly stained filamentous structures. (B) KOH mount demonstrating abundant septate, hyaline hyphae with branching KOH: potassium hydroxide

Corneal scrapings were obtained under aseptic precautions and inoculated into 5% sheep blood agar and Sabouraud dextrose agar (SDA) plate along with direct microscopy for 10% potassium hydroxide (KOH) mount. A provisional diagnosis of fungal keratitis was made, and empirical therapy was initiated with natamycin 5% eye drops hourly, homatropine 2% thrice daily, and lubricants to relieve ciliary spasm and protect the ocular surface. The findings by direct microscopy are as follows: Gram staining revealed faintly stained filamentous structures (Figure 2A), and KOH mount showed abundant septate hyaline hyphae with branching (Figure 2B).

**Figure 3:**
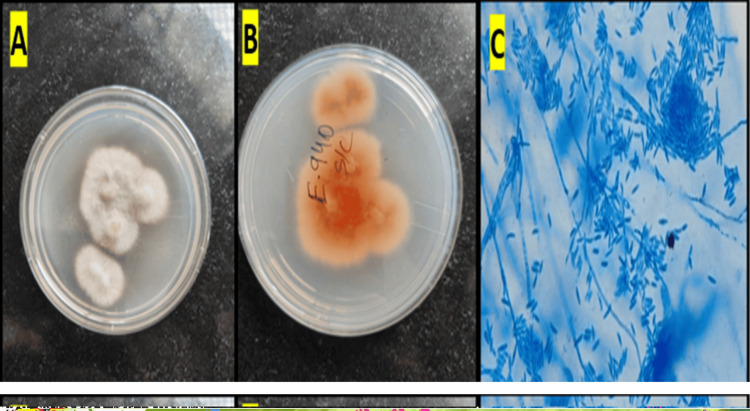
Growth of Fusarium solani on SDA and microscopic morphology (A,B) White, fluffy colonies seen on SDA. (C) LPCB mount showing hyaline, septate hyphae with characteristic canoe‑shaped macroconidia SDA: Sabouraud dextrose agar; LPCB: lactophenol cotton blue

Cultures on SDA and blood agar demonstrated white, fluffy colonies by day 3 (Figures 3A, 3B). A lactophenol cotton blue mount from the culture showed hyaline, septate hyphae with characteristic canoe-shaped macroconidia, confirming Fusarium species (Figure 3C) and later confirmed by matrix-assisted laser desorption/ionization time-of-flight mass spectrometry to be *Fusarium solani.*

Following microbiological confirmation, the treatment regimen was escalated to dual antifungal therapy with natamycin 5% and voriconazole 1% topical drops. By the third day of antifungal therapy, subjective improvement in vision and reduced discomfort were reported. Slit-lamp reevaluation showed resolution of hypopyon, reduction of stromal edema, and a decrease of the epithelial defect to about 1 mm, with fluorescein staining limited to the ulcer margins. Over the subsequent week, the ulcer demonstrated steady healing, with complete resolution of the epithelial defect and no evidence of stromal thinning, melting, or secondary infection.

At follow-up, corneal clarity had significantly improved, slit-lamp examination appeared normal, and the child's visual acuity had recovered to 6/6 bilaterally, with no surgical intervention required.

## Discussion

Fungal keratitis is a major cause of corneal blindness worldwide, with a disproportionately high burden in tropical and subtropical regions. The estimated global incidence of infectious keratitis is 23.6 per 100,000 people annually, but this rises sharply in countries with hot, humid climates and agrarian risk factors, reaching 113 per 100,000/year in South India, 339 in Bhutan, 710 in Burma, and 799 in Nepal [6]. By contrast, developed countries such as the United States, England, and Australia report much lower rates, ranging from 6.6 to 40.3 per 100,000/year, where contact lens use rather than trauma is the predominant risk factor [6].

Fungal pathogens contribute to nearly one-quarter of all microbial keratitis cases globally, with India reporting even higher rates of 26%-37% of corneal ulcers being fungal in origin [6]. Among these, Fusarium species dominate in tropical climates, accounting for 40%-60% of fungal keratitis cases [6-8]. The estimated global burden of fungal keratitis exceeds one million cases annually, with Fusarium keratitis alone responsible for 420,000-630,000 cases [8]. This aggressive pathogen is distinguished by rapid stromal invasion, poor responsiveness to conventional antifungals, and a frequent need for surgical intervention.

**Figure 4:**
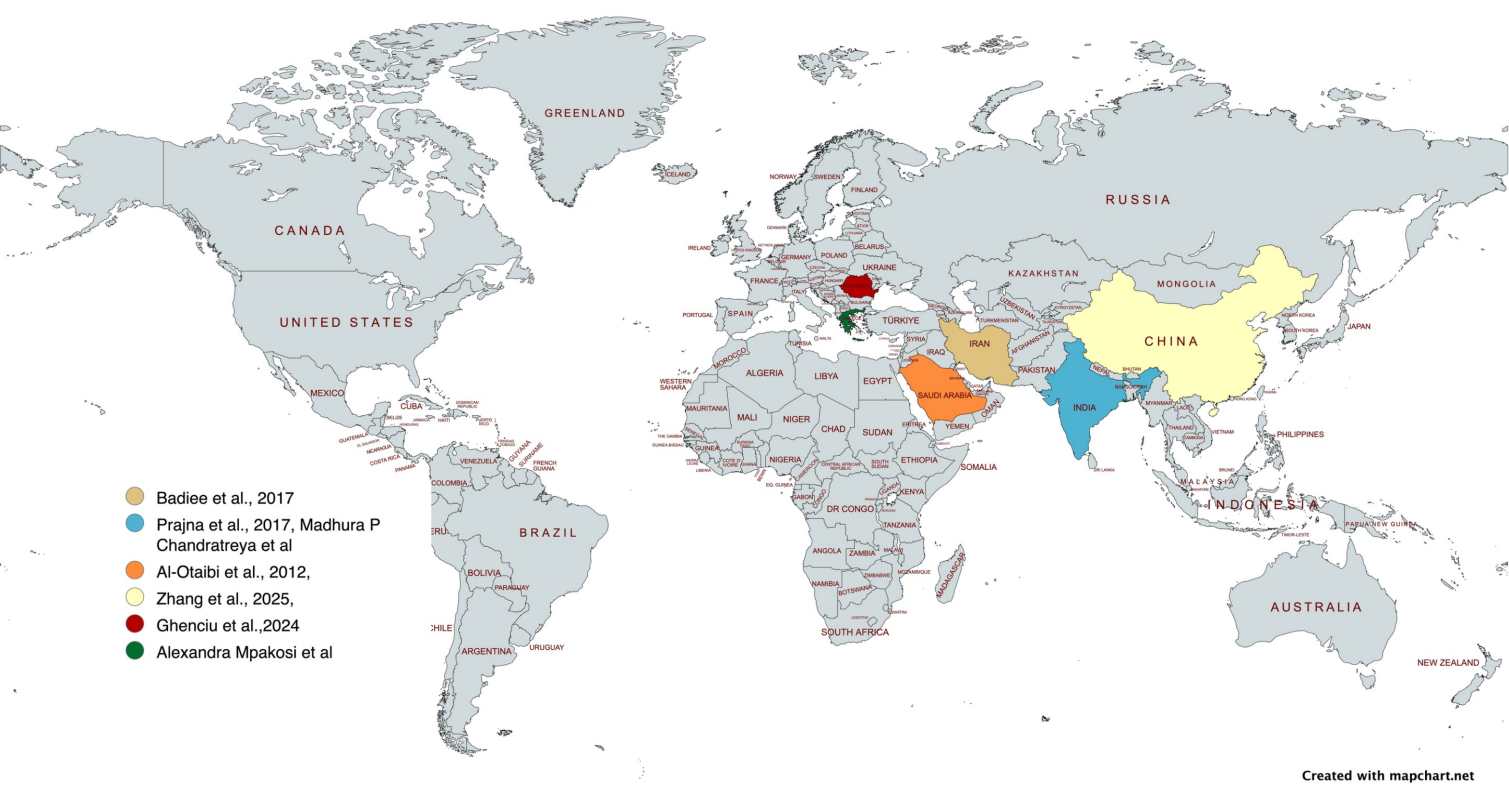
Published cases of pediatric Fusarium keratitis in different countries Geographic distribution of pediatric Fusarium keratitis cases [1,9,12-15] Source: Map created using MapChart (www.mapchart.net)

Although adults constitute the majority of keratitis cases (87%-97%), children represent a smaller but clinically important group, comprising 3%-13% of all keratitis presentations [9-11]. Pediatric keratitis is often associated with trauma in developing countries and contact lens use in developed regions [9,11]. The course is frequently more severe in children due to delayed recognition, communication barriers, and challenges with compliance, which increase the risk of amblyopia and long-term visual disability [9,11]. Within pediatric fungal keratitis, Fusarium species are consistently reported as the most common isolate, comprising more than 60% of cases in large series [9,11] (Figure 4 and Table 1).

**Table 1:** Global epidemiology of pediatric Fusarium keratitis PCR: polymerase chain reaction; CL: contact lens; PK: penetrating keratoplasty; LKP: lamellar keratoplasty; NLR: neutrophil-to-lymphocyte ratio; TPK: therapeutic penetrating keratoplasty

Study (region)	Year	Population	n (Fusarium)	Main risk factors	Diagnosis (molecular)	Monotherapy, n (%)	Dual Therapy (n, %)	Surgery (n, %)	Resolved on medical, n (%)	Main antifungals	Key outcomes
Al-Otaibi [9] (Saudi Arabia)	2012	Pediatric	34	Trauma	Culture	Not specified	Not specified	12 (35.3%)	22 (64.7%)	Natamycin, voriconazole	Early diagnosis, intensive therapy, and surgery improve outcomes
Badiee et al. [12]	2017	Pediatric	Review	Trauma	Culture	Not specified	Not specified	Not specified	Not specified	Natamycin, voriconazole	Early diagnosis and intensive therapy critical
Prajna et al. [1] (India)	2017	Pediatric	234 (all pediatric keratitis)	Trauma	Culture	Not specified	Not specified	Not specified	Not specified	Natamycin	Fusarium is the most common fungus in children
Ghenciu et al. [13]	2024	Mixed	Review	Trauma, CL use	Culture, molecular	Not specified	Not specified	Not specified	Not specified	Natamycin, voriconazole	Polyenes and azoles essential; molecular advances emerging
Zhang et al. [14] (China)	2025	Pediatric	47 children (48 eyes)	Trauma (dust/vegetative)	Culture, PCR	8/48 (16.7%)	Not specified	32/48 (66.7%) (21 PK, 11 LKP)	8/48 (16.7%)	Voriconazole, natamycin	Fusarium 41.7%; ulcer size predicts surgery; NLR predicts risk
Cintra et al. [16]	1969-2023	Mixed	Review	Contact lens usage	Culture	Not specified	Not specified	Not specified	Not specified	Natamycin, voriconazole	Timely identification and early initiation of antifungal treatment
Mpakosi et al. [15] (Greece)	16 years	Mixed	35 fungal keratitis Fusarium species (n = 21, 61.8%)	Corneal injury by plant material and soft contact lens	Culture	Not specified	Not specified	Keratoplasty was required in 40% and enucleation in 8%	Antifungal therapy alone was 52%	Voriconazole, liposomal AmB	A large proportion of cases resulted in keratoplasty despite appropriate antifungal treatment
Chandratreya et al. [3] (South India)	18 months	Pediatric	6.8% of eyes were culture positive, with bacterial isolates in 17.9% and fungi in 82.1%. Fusarium species (67.8%)	Trauma (unidentified foreign body)	Not specified	Polytherapy	Not specified	TPK was required for 2.6% of eyes	87.80%	Natamycin and 0.5% moxifloxacin concurrently	Trauma was the leading predisposing factor for microbial keratitis

The present case highlights these global patterns: classic clinical features, including feathery-edged infiltrates, stromal involvement, and early hypopyon-facilitated timely suspicion of fungal keratitis. Importantly, bacterial and fungal keratitis are often clinically indistinguishable at presentation, making early microbiological evaluation indispensable. Turnaround time from corneal scraping to definitive identification was three days, aligning with recommendations in the literature for timely confirmation and targeted therapy [17].

Dual antifungal therapy with natamycin and voriconazole was employed, reflecting evidence that combination therapy can be more effective than monotherapy in Fusarium infections [18]. Natamycin remains the gold-standard first-line agent for filamentous fungi, while voriconazole, with its superior stromal penetration, is valuable in deep or refractory infections [19]. The rapid resolution of hypopyon and reduction in ulcer size within 72 hours mirror reported outcomes of early, aggressive dual therapy.

This case also highlights the detrimental role of unsupervised corticosteroid use in ocular disease. Steroids, though beneficial in immune-mediated conditions, suppress local defenses and permit unchecked fungal proliferation [5]. Public health interventions, pharmacist regulation, and awareness campaigns are essential to prevent such misuse, particularly in low-resource settings. While pediatric Fusarium keratitis remains relatively rare, outcomes are often poor due to late diagnosis, inadequate therapy, and surgical dependence. By contrast, the favorable resolution in this case illustrates how early clinical recognition, prompt microbiological confirmation, and aggressive anti-fungal therapy can dramatically improve prognosis. At a global level, however, challenges, including limited access to natamycin in many regions, suboptimal drug delivery systems, and high surgical burden, remain. These underscore the urgent need for novel antifungal agents, improved treatment delivery, and preventive strategies to reduce the global burden of Fusarium keratitis.

## Conclusions

Pediatric Fusarium keratitis, though rare, demands high clinical suspicion following ocular trauma. This case highlights the effectiveness of early dual antifungal therapy, initiated based on classical clinical signs and rapidly supported by microbiological evidence. The favorable outcome without surgical intervention reinforces the importance of early sample collection, accurate diagnosis, and intensive medical management. It also serves as a reminder of the potential hazards of unsupervised corticosteroid use in red eye conditions. This case contributes valuable evidence to the limited literature on pediatric fungal keratitis and supports current recommendations advocating prompt empirical treatment when fungal etiology is suspected.
